# Rare Coexistence of Acute Monoblastic Leukemia with Chronic Lymphocytic Leukemia

**DOI:** 10.1155/2018/6452843

**Published:** 2018-11-06

**Authors:** Vikrant Singh Bhar, Vasudha Gupta, Mahak Sharma, Rishi Dhawan, Shilpi Modi, Mona Vijyaran

**Affiliations:** ^1^Associate Consultant, Department of Haematopathology, Artemis Hospitals, Gurgaon, India; ^2^Senior Resident, Department of Haematopathology, Artemis Hospitals, Gurgaon, India; ^3^Assistant Professor, Department of Hematology, AIIMS, New Delhi, India; ^4^Associate Consultant, Department of Histopathology, Artemis Hospitals, Gurgaon, India; ^5^Associate Consultant, Department of Hematooncology, Artemis Hospitals, Gurgaon, India

## Abstract

Acute monoblastic leukemia (AMoL) is a rare hematopoietic neoplasm, and simultaneous occurrence of acute monoblastic leukemia with chronic lymphocytic leukemia is very rare and only a few cases have been reported in the literature. We here report a rare case of dual hematological malignancy in an 85-year-old male. The peripheral blood and bone marrow examination revealed dual population of atypical cells, comprising large cells with opened-up chromatin having monocytic appearance and small mature-appearing lymphocytes. Flowcytometric immunophenotyping confirmed the monocytic lineage of cells, whereas small lymphocytes showed the immunophenotype consistent with chronic lymphocytic leukemia (CLL). The final diagnosis was made as acute monoblastic leukemia with associated CLL. This is a rare case scenario, and this highlights the importance of careful morphological examination and flowcytometric immunophenotyping in the exact characterization of hematopoietic malignancies.

## 1. Introduction

Dual hematological malignancy occurring simultaneously in a patient is a rare phenomenon. There are many case reports in literature describing dual coexistence of CLL with other hematological disorders. Brouet et al. reported eleven cases of CLL with coexistent multiple myeloma. Six of these cases expressed different immunoglobulins, suggesting biclonal nature of the disease [1]. Similar cases of dual coexistence of CLL with multiple myeloma have been reported in the past [2]. Coexistence of CLL with acute myeloid leukemia is even rarer, and only a few case reports have been reported so far. Carulli et al. have reported a case of acute myeloid leukemia with monoblastic features associated with CLL [3]. Gottardi et al. reported a similar case of acute myeloid leukemia with maturation and CLL. Authors also did clonal studies and showed that both diseases represent two different clones [4]. Lai et al. reported five cases of untreated CLL with acute myelogenous leukemia and myelodysplastic syndrome [5]. Apart from de-novo occurrence of concomitant CLL and acute myeloid leukemia, patients with CLL can rarely show transformation to acute myeloid leukemia (AML). Hatoum et al. in their review of literature found only 6 cases of CLL transforming to AML [6]. We here report such a rare case of dual hematological disorder, acute monoblastic leukemia with chronic lymphocytic leukemia confirmed by flowcytometric immunophenotyping.

## 2. Case Report

We here report a case of an 85-year-old male who was apparently well 15 days back, when he started developing swelling of bilateral feet. The patient also complained of decreased urine output with poor urinary stream. The patient has a history of breathlessness, more so on exertion. The patient is an ex-smoker and has a history of loss of appetite and loss of weight since 1-2 months. Also, there is a history of anemia in the past with a recorded haemoglobin (Hb) level of 78 g/l. The patient's clinical examination showed multiple, nontender firm lymph nodes in the right upper jugular, middle jugular, right and left submandibular, and multiple right-sided axillary lymph nodes. His complete blood count parameters were as follows: Hb, 58 g/l; platelet count, 63 × 10^9^/l; and total leukocyte count (TLC), 230 × 10^9^/l. Differential counts on peripheral blood smear (PBS) were as follows: blasts, 30%; promonocytes, 5%; monocytes, 5%; neutrophils, 3%; and lymphocytes, 57%. Lymphocytes appeared mature with many smudge cells. Clinical and laboratory features of the patient were consistent with tumor lysis syndrome (TLS). Laboratory parameters supporting TLS were as follows: uric acid, 11.5 mg/dl; calcium, 7.7 mg/dl; phosphorus, 4.8 mg/dl; potassium, 4.2 meq/L;and serum creatinine, 2.42 mg/dl.

**Figure 1:**
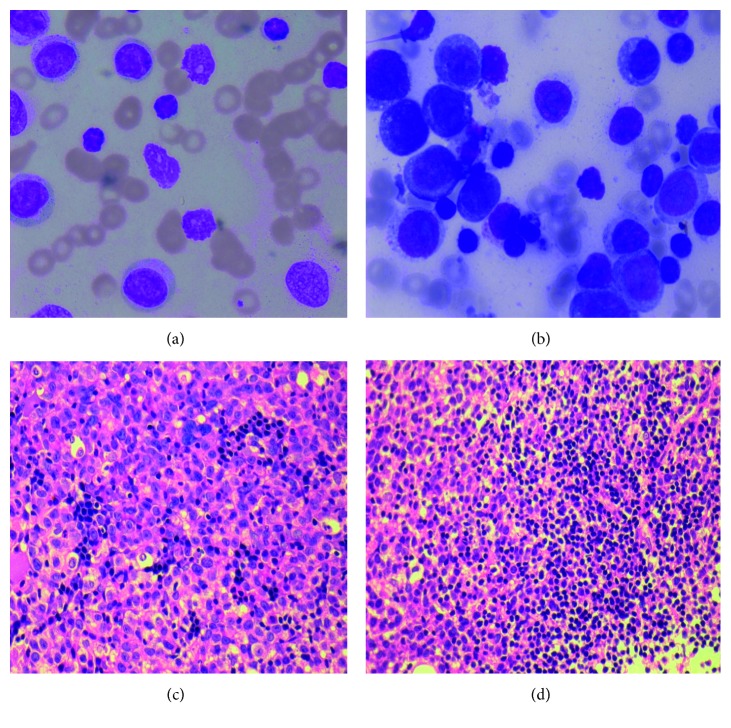
(a, b) Peripheral blood film and bone marrow aspirate showing dual population (×40, May Grunwald Giemsa). (c, d) Bone biopsy with sheets of immature cells with interstitial infiltration and nodular aggregates of lymphocytes, respectively (×40, H&E).

Bone marrow examination showed markedly hypercellular smears with reduced megakaryocytes and erythropoiesis. Bone marrow differential counts are summarized in Table 1. Bone marrow biopsy was markedly hypercellular with sheets of immature cells with abundant cytoplasm (monocytic look) replacing normal hematopoietic elements. In addition, there were interstitial increase and intertrabecular small to large collections of mature lymphocytes. Representative pictures of peripheral blood and bone marrow findings are compiled in Figure 1.

**Figure 2:**
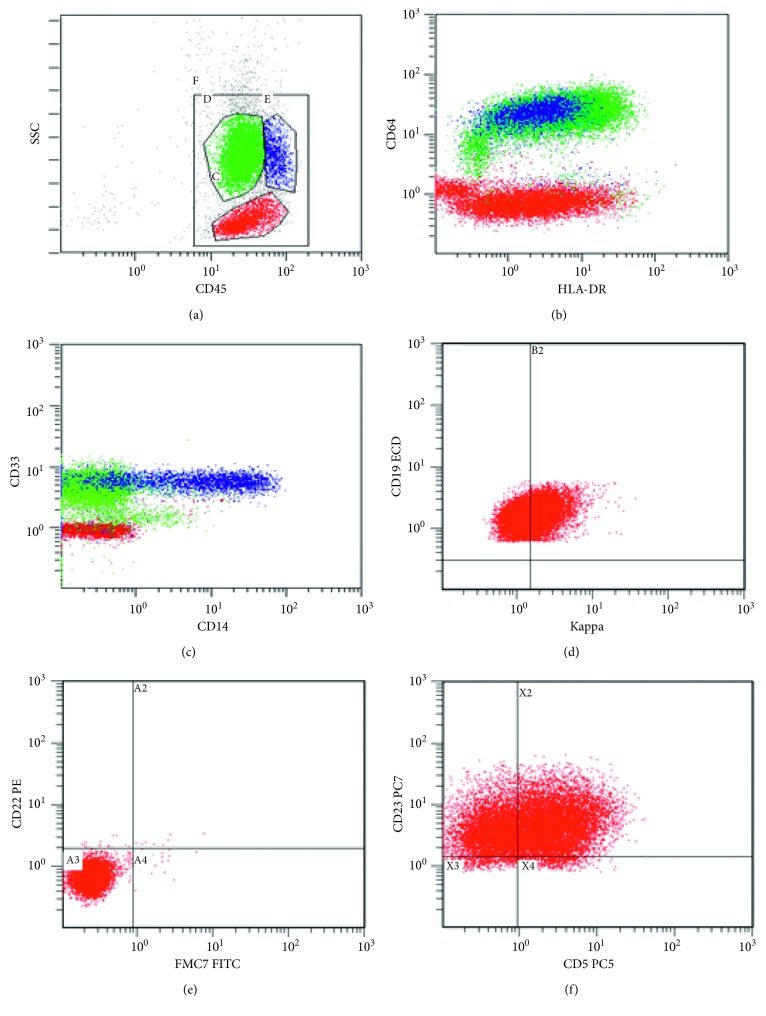
(a) CD45 versus side scatter (SSC) dot plot showing three distinct populations. Lymphocytes: CD45 positive and low side scatter (red color). Monocytic precursors and monocytes: CD45 positive and moderate side scatter (green and blue color, respectively). (b, c) Population in green and blue color was positive for CD64, CD33, and HLA-DR. More mature cells were also positive for CD14. (d–f) Lymphocytes were predominantly B cells and showed dual positivity for CD5 and CD23 with dim kappa and negative FMC7 and CD22.

PBS and bone marrow aspirate lymphocytosis made us to suspect a dual disorder, and we put a combined flowcytometry panel for acute leukemia and chronic lymphoproliferative disorder. Gating was done on CD45 versus the side scatter plot. The gating plot revealed two different populations, CD45 positive with moderate side scatter (Population 1-monoblasts) and CD45 positive with low side scatter (Population 2-lymphocytes). Population 1 showed monocytic markers with negative MPO, B-lineage, and T-lineage markers. Population 2 was positive for CD19 and showed dual positivity for CD5 and CD23. Overall immunophenotyping features were consistent with acute leukemia with monocytic differentiation and chronic lymphocytic leukemia. Immunophenotyping features are compiled in Table 2 and Figure 2.

The patient was explained about treatment options and prognosis, and he refused to undergo any further investigations and therapy.

## 3. Discussion

The B-cell non-Hodgkin lymphoma (B-cell NHL) makes up to 80–85% of all NHLs in India. Diffuse large B-cell lymphoma (DLBCL) is the commonest type of B-cell NHL. The prevalence of CLL of all NHL cases in India has been reported to be around 4-5% as compared to the much higher prevalence of CLL from Western countries [7].

This is a rare case of simultaneous presentation of untreated CLL with acute myeloid leukemia with monoblastic differentiation. Similar rare case reports of dual hematological malignancy have been reported in the past. In these case reports, CLL is a common partner with the second disease being plasma cell neoplasm, acute myeloid leukemia, or myelodysplastic syndrome [1–5]. The mean age of patients was 73.8 and 67.2 years in the case series reported by Brouet et al. [1] and Lai et al. [5]. Our patient was 85 years old indicating the fact that chances of having dual malignancy are higher in elderly patients as chances of acquiring plasma cells neoplasms, CLL increases, and other hematological malignancies increases with age. The peripheral smear of the patient showed distinct double population of small mature-appearing lymphocytes and large immature cells with monocytic morphological features. In addition, smudge cells were seen. Bone marrow examination also showed blasts with the second population of small lymphocytes. The review by Kotchetkov et al. at their center of 3,036 patients revealed 41 cases of synchronous dual hematological malignancy (SDHM). The authors divided SDHM cases in three groups based on the type of combination. The myeloid/lymphoid group revealed monoclonal gammopathy of unknown significance (MGUS) as the most common hematological malignancy occurring with myeloid neoplasms. This study did not find combination of CLL with AMoL [8]. The present case here represents a rare myeloid/lymphoid type of SDHM. This case highlighted the importance of careful morphological examination and deciding immunophenotyping panels as guided by morphological findings. Similarly, a close careful follow-up of the cases on therapy might help in picking up cases of CLL transforming to AML as has been rarely reported in the literature [6]. The systemic approach not only helps in exact characterization of the disease but also saves time, labour, and reagents. Flowcytometry immunophenotyping is indispensable for exact typing and confirmation of the disease. So, a high index of suspicion, careful morphological examination, and flowcytometric immunophenotyping are required to diagnose rare case presentations.

## Figures and Tables

**Table 1:** Findings of bone marrow aspirates.

Blast (%)	Promonocyte (%)	Metamyelocyte (%)	Lymphocyte (%)	Monocytes (%)	Erythroid precursor (%)
54	02	01	40	01	02

**Table 2:** Immunophenotyping results of large cells (Population 1) and small cells (Population 2).

*Population 1: CD45 positive with moderate side scatter*	
Positive markers	CD33, CD64, CD14(Only in minor subset with bright CD45), HLA-DR, dim CD13
Negative markers	CD19, CD3, CD5, CD10, cyCD79a, CD22, MPO, CD34, TdT, CD117

*Population 2: CD45 positive with low side scatter*	
Positive markers	CD19, cyCD79a, dim CD20, CD5, CD23, CD200, dim kappa
Negative markers	CD3, myeloid and monocytic markers, CD22, FMC7, CD10, immaturity markers, lambda
